# Bad for the Temperance Cause

**Published:** 1880-12

**Authors:** 


### BAD FOR THE TEMPERANCE CAUSE.

An article has been extensively published of late in the secular press concerning the theories of Dr. Geo. M. Beard to the effect that Americans, as a nation, do not drink enough. The avidity with which such an item is read, is an indication of the morbid propensity many persons have to conform their diet to some imaginary standard. Whether one doctor teaches by example that all men should fast, or another advocates by lecturing that all should drink copiously, both are sure of attracting public attention. Indeed, to judge from the extracts given one might imagine that just before Dr. Beard's lecture, he too, added practice to precept by frequent and liberal libations. At any rate, if large quantities of fluid food are good for the nerves, as the writer would have us believe, we can at least rest assured that one or two of our acquaintances will never need the services of any neurologist. It is probably the fact, however, that unwarranted deductions have been drawn from a garbled account of Dr. Beard's views on this matter. He is, of course, not responsible for this, and it is only one of the annoyances from misinterpretation, to which medical men are continually subjected.

